# From guidelines to the bedside: a mobile decision-support and documentation tool for adult advanced life support

**DOI:** 10.1016/j.resplu.2026.101476

**Published:** 2026-09-06

**Authors:** Mehmet Bahadır Savaş, Tülay Dikencik, Füsun Özkaya, Yusuf Doğan, Ayşe Yılmaz

**Affiliations:** Department of Anestehesiology and Reanimation, Tinaztepe University, Faculty of Medicine, İzmir 35270, Turkey

To Editor,

We read with great interest the 2025 European Resuscitation Council (ERC) Guidelines on Education for Resuscitation by Nabecker et al.^1^ The framework progressing from scientific evidence through teaching and learning to implementation and ultimately to best clinical practice and improved neurological outcomes highlights a persistent challenge in everyday practice: the gap between guideline recommendations and what resuscitation teams can deliver under the extreme cognitive load of cardiac arrest.

This gap is also evident in digital resuscitation tools. In a systematic review by Fijačko et al.,^3^ only 6 of 1207 candidate applications (0.49%) designed to teach basic life support met predefined quality criteria. Similarly, a controlled simulation study showed that a dynamic mobile application improved residents’ adherence to ACLS guidelines, compression fraction, and correct interventions,^4^ although its benefit in real in-hospital resuscitation remained unvalidated.

To address this implementation gap, we developed a mobile decision-support and documentation application designed primarily for real-time bedside use during cardiac arrest. The application translates the ERC 2025 Adult Advanced Life Support (ALS) algorithm^2^ into an interactive, time-critical workflow. A two-minute cycle timer is synchronized with a 120/min metronome. Adrenaline reminders are triggered every 3–5 min, while amiodarone reminders are linked to cumulative shock number. The refractory VF pathway, including transition to anteroposterior pad placement, is activated after three consecutive unsuccessful shocks. Medication prompts are linked to confirmed intravenous or intraosseous access, with alternative-route guidance when access is unsuccessful Eleven special-circumstance protocols, including anaphylaxis, hypothermia, pulmonary embolism, and maternal cardiac arrest, are integrated directly into the arrest workflow. Interventions can be time-stamped and documented in Utstein.

Beyond guideline reminders, the application reduces decision latency by shifting recall to recognition, enables real-time structured data capture, automates Utstein documentation, and supports education through replay and debriefing.

The interface was specifically designed for the resuscitation environment, incorporating glove-compatible touch targets, audible confirmation, double confirmation for irreversible actions, and high-contrast, glance-readable displays.

We are currently preparing a simulation-based evaluation comparing guideline adherence, medication-timing accuracy, and documentation completeness with and without the application. If successful, this approach could provide a practical means of bringing the ERC 2025 implementation layer directly to the bedside and supporting more consistent delivery of guideline-based resuscitation.

We congratulate the guideline authors for articulating this implementation-focused vision and would welcome collaboration with the ERC education and implementation community to further develop and evaluate such tools.

## CRediT authorship contribution statement

**Mehmet Bahadır Savaş:** Conceptualization, Data curation, Formal analysis, Methodology, Supervision, Writing – original draft. **Tülay Dikencik:** Data curation, Formal analysis, Methodology, Writing – original draft. **Füsun Özkaya:** Data curation, Formal analysis, Methodology, Writing – original draft. **Yusuf Doğan:** Data curation, Formal analysis, Methodology, Writing – original draft. **Ayşe Yılmaz:** Data curation, Formal analysis, Methodology, Supervision, Writing – original draft.

## Funding

None.

## Declaration of competing interest

The authors declare no conflict of interest. The application described was developed by the corresponding author (M.B.S.); it is non-commercial and no revenue is derived from it.
